# A comparative study of the microtensile bond strength and microstructural differences between sclerotic and Normal dentine after surface pretreatment

**DOI:** 10.1186/s12903-019-0899-x

**Published:** 2019-10-07

**Authors:** Jinhua Wang, Weijian Song, Lei Zhu, Xin Wei

**Affiliations:** 1 Jiangsu key Laboratory of Oral Diseases, Department of Conservative Dentistry and Endodontics, Stomatological Hospital, Nanjing Medical University, Nanjing, China; 2 The Affiliated Suzhou Science and Technology Town Hospital of Nanjing Medical University, Suzhou, China

**Keywords:** Non-caries sclerotic dentin, Surface pretreatment, Micro-tensile bond strength, Microstructure, Self-etching adhesive

## Abstract

**Background:**

The resin bond strength of sclerotic dentine is significantly lower than that of the normal dentine, which paused a challenge for bonding procedures clinically. The aim of this study was to compare the effects of different surface pretreatments on the micro-tensile bond strength and microstructure between sclerotic dentine and normal dentine.

**Methods:**

Eighty teeth that were collected, forty premolars with typical wedge-shaped defects visually graded as class III were assigned as the sclerotic dentine group (SD), the other forty normal premolars with artificial wedge-shaped defects were assigned as the normal dentine group (ND). Each group was randomly subdivided into eight subgroups according to the solution used: 35% phosphoric acid, 15% EDTA, 5% or 10% NaClO. Then the dentine surface was examined using a scanning electron microscope (SEM). The lesions were restored using self-etching adhesive and the subsequent resin composite. The teeth were sectioned into sticks for the micro-tensile bond strength analysis, and the data were analysed using the SPSS17.0 software package (α = 0.05).

**Results:**

First, for the ND groups, after pretreatment using 35% phosphoric acid, and 35% phosphoric acid + 5% or 10% sodium hypochlorite, the bonding strengths of the normal dentine were higher than that of the other groups (*P <* 0.05). Second, for the SD groups, after pretreatment using 35% phosphoric acid, 15% EDTA, and 35% phosphoric acid + 5% or 10% sodium hypochlorite, the bonding strengths of the sclerotic dentine were higher than that of the other groups (*P <* 0.05). Third, the bond strengths of the sclerotic dentine were lower than that of the normal dentine without any pretreatment (*P* < 0.05). After pretreatment using 35% phosphoric acid + 5% or 10% sodium hypochlorite, the bonding strengths of the sclerotic dentine were higher than that of the normal dentine (*P* < 0.05). SEM observation showed that the appearances of dentine surface were changed after pretreatment using the above solutions, with the reduced smear layer, opened small groove and increased dentinal tubules.

**Conclusion:**

Pretreatment of dentine using 35% phosphoric acid+ 5% or + 10% sodium hypochlorite changed the microstructure of the sclerotic dentine surface and subsequently increased the micro-tensile bond strength.

## Background

Over a lifetime, chewing, tooth brushing and other mechanical friction can lead to the defects in dentine tissue [1]. Defects in cervical dentin combined with occlusal stresses, chronic stimuli of a low intensity and high frequency, bacterial colonization and a defensive change in the dentine itself, gradually caused to the loss of teeth substances [2, 3]. The non-carious sclerotic dentine was formed subsequently along the cervical enamel junction.

Bonding resin composite restorations without cavity preparation are provided to restore teeth under this condition. However, several reports have indicated that the resin bond strength of non-carious sclerotic dentine is significantly lower than that to normal dentine [4, 5]. Frequent falling off the resin filling from the sclerotic dentine leads to a higher rates of restoration failure, which takes the loss of time and money for both patients and dentists [6].

The bonding difficulty between the non-carious sclerotic dentine and filling resin raises a series of problems in the clinic. For one side, sclerotic dentine is an abnormal bonding substrate that exhibits a high degree of variability, in terms of both dentinal tubule obliteration by mineral salts and the thickness of the surface hyper-mineralized layer [7]. On the other side, bacteria can be detected on the top of hyper-mineralized layer or embedded in a partially mineralized matrix [7]. The existence of bacteria and mineralized bacterial matrices, as well as the hypermineralized surface layer and mineral-occluded tubules, causes the sclerotic dentine to be a distinct multi-layered bonding substrate [8]. These above physiological and pathological alterations of the sclerotic dentine prevent the hybridization of the underlying sclerotic dentin to the filling resin [9–13], and also decrease the bonding strength between the resin and dentine.

The basic bonding mechanism of the adhesion between resin and dentin is an exchange process involving the replacement of the minerals removed from the hard dental tissue by resin monomers, which, upon setting, become micromechanically interlocked in the created porosities. Presently, two bonding mechanisms with resin monomers are widely used with modern adhesive systems: total-etching (etch and rinse) and self-etch approaches [14, 15]. Several studies have reported that there is no marked difference between the self-etching and total-etching adhesive systems of sclerotic dentine in terms of bond strength [5, 16], whereas other studies have found a lower bonding strength to sclerotic dentine using the self-etch approach than using the total-etching system [17, 18]. In addition, other studies suggested that the lower bonding strength could be improved to a certain extent by changing the protocols relevant to the adhesion [19]. For example, roughening the surface of sclerotic dentine with diamond burs increases the hybrid layer thickness [17], and increasing the acid conditioning time can also improve resin bonding to sclerotic dentine [20]. However, the above protocols still could not change a higher incidence of restoration failure.

As those previous methods of cementing of sclerotic dentine are still controversial, the aim of this in vitro study was to evaluate the effects of different surface treatments on sclerotic dentine of the human teeth in terms of the bond strength and microstructure using a self-etching adhesive system, Adper™ Easy One. Meanwhile, the effects of the pretreatment on the dentine strength were analysed using a micro-tensile bonding test, and that on microstructure of the dentine surface were also observed using a scanning electron microscope (SEM). The goal of the study was to find a modified protocol that could improve the adhesion between sclerotic dentine and resin, which will facilitate the clinical treatment of wedge- shaped defects formed by sclerotic dentine. The null hypothesis was that there would be no difference of the micro-tensile bond strength and micro-structure between sclerotic dentine and normal dentine after the different surface pretreatments.

## Methods

### Sampling and sample preparation

The local ethical Committee Department of Stomatological Hospital of Nanjing Medical University approved this study (Institutional Review Board) (IRB; approval number PJ 2015–050-001). In addition, patients were requested if they could provide their teeth for this research. Informed consent form was obtained and signed from patients when they gave consent. Eighty human premolars extracted for periodontal reasons or via orthodontic extraction were collected. Plaque and tartar were removed from all of the extracted teeth using an ultrasonic scaler. Forty premolars with typical wedge- shaped defects, which were visually graded as class III according to the North Carolina dentine hardening grading (Table 1), were defined as the sclerotic dentine group (S group) [21]. The other forty teeth were normal premolars with artificial wedge-shaped defects and were defined as the normal dentine group (N group). The teeth were stored in 5 ml/L chloramine-T solution at 4 °C for no longer than 1 month before being used in the experiment.

**Table 1:** Sclerosis Scale (modified)

Category	Description
I	No sclerosis present. Dentin is light yellow or whitish in color with little discoloration. Dentin is opaque, with little translucency or transparency. These lesions are typical in young individuals.
II	More than category I, but less than 50% of the difference between categories I and IV.
III	Less than category IV, but more than 50% of the difference between categories I and IV.
IV	Significant sclerosis present. Dentin is dark yellow or even discolored (brownish). Dentin appears glassy, with significant translucency or transparency evident. These lesions are typical in older individuals.

Artificial wedge-shaped lesions were prepared on intact teeth in the buccal cervical region by means of a fine-grit cone-shape diamond bur (TR-11F, MANI DIA-BURS; mean particle size 53–63 μm), which was mounted in a high-speed handpiece under copious air-water cooling. The shape and size of the artificial cavities were as follows: mesiodistal width of 4.0 mm, buccogingival height of 4.0 mm and maximum depth of 3.0 mm [22]. The artificial cavities were similar to natural ones. Afterward, the teeth with natural and artificial cervical lesions were cleaned with alcohol pellets and subjected to 10 s of water spray.

In addition, the hardness, colors, and tooth position of the isolated teeth in the two groups were paired using the balanced line test.

### Experimental groups and pretreatments

The sclerotic dentine group and the normal dentine group were each randomly subdivided into eight groups according to the different pretreatment methods (Table 2).

**Table 2:** Surface pretreatments of dentine

Pretreatment	method
M1	distilled water
M2	35% phosphoric acid 30 s
M3	5% NaClO solution 60 s
M4	15% EDTA gel 60 s
M5	15% EDTA gel 60 s, 5% NaClO solution 60 s
M6	15% EDTA gel 60 s, 10% NaClO solution 60 s
M7	35% phosphoric acid 30 s, 5% NaClO solution 60 s
M8	35% phosphoric acid 30 s, 10% NaClO solution 60 s

M: Pretreatment methods; M1-M8: the different pretreatment methods.

The descriptions of the studied groups were as follows: N1-N8 normal dentine group (*n* = 4 per group) and S1-S8 sclerotic dentine group (*n =* 4 per group) (Table 3). N1 and S1 were used as a pair of control groups for the normal and sclerotic dentine groups, respectively.

**Table 3:** The experimental groups

Group	Treatment
N1, S1	distilled water
N2, S2	35% phosphoric acid 30 s
N3, S3	5% NaClO solution 60 s
N4, S4	15% EDTA gel 60 s
N5, S5	15% EDTA gel 60 s, 5% NaClO solution 60 s
N6, S6	15% EDTA gel 60 s, 10% NaClO solution 60 s
N7, S7	35% phosphoric acid 30 s, 5% NaClO solution 60 s
N8, S8	35% phosphoric acid 30 s, 10% NaClO solution 60 s

Note: N: Normal dentine; S: Sclerotic dentine

### Sample restoration

Following pretreatment, the self-etching adhesive Adper™ EasyOne (3 M ESPE, USA) adhesive system was applied according to the manufacturer’s instructions. Use a brush full of adhesive to apply it evenly over the dentine surface of the wedge-shaped defect for 20 s, gently dry the tooth surface for 5 s, and light cured it for 10 s. Next, all teeth received a hybrid composite restoration (3 M Filtek Z250 (A3), 3 M ESPE, USA) in two increments of 2 mm to form a raised cylinder [4, 5]. Then the composite restoration were light cured for 40 s using a halogen light-curing unit set at 400 mW/cm^2^ (WOODPECKER, Guilin, China), as conventionally performed for the micro-tensile bond strength test [17, 23]. According to the advice of ISO, the specimens were stored at 37 °C in water for 24 h.

### Micro-tensile bond strength test (μ-TBS)

**Fig. 1:**
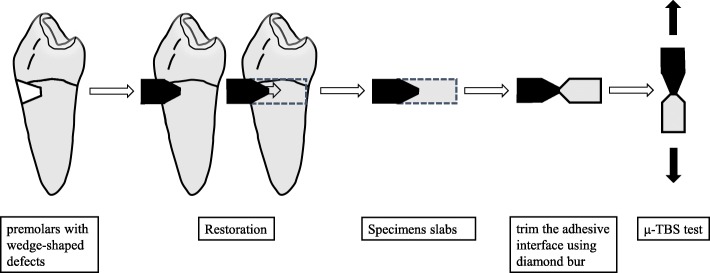
Preparation of specimen for the micro-tensile test

The roots and coronal tissue were removed with a low-speed diamond saw (Isomet, Buehler Ltd., USA). The specimens were sectioned bucco-lingually to obtain three or four slabs with a length of 8 mm, width of 4 mm, and thickness of 1 mm for micro-tensile testing (μ-TBS), and each group contained 10 testing specimens. Then, the slabs were trimmed to an hourglass shape using a regular diamond bur (TF13/TF11, MANI, Japan), and the narrowest portion of the cross-sectional dimensions at the adhesive interface of each specimen was approximately 1.0 mm^2^ [22] (Fig. 1). The final width and thickness of the bonded area were calculated to the nearest 0.01 mm by means of a digital micrometre (Shanghai, China).

All specimens were glued to a Geraldelli jig with cyanoacrylate adhesive and pulled in tension (Micro-tensile tester, Isomet, USA) at a cross head speed of 1 mm/min until failure [24, 25]. Bond strengths were calculated by dividing the load at failure by the cross-sectional bonding area.

### The specimen mode of failure

Using a stereoscopic microscope at 10× magnification (Leica, Germany), the specimen fracture mode was classified as three kinds: (1) adhesive failure (80–100% of the failure occurred at the resin-dentine bond interface); (2) mixed failure (mixed with adhesive failure between resin and dentine, and cohesive failure in resin and/or dentine); (3) cohesive failure in substrate (80–100% of the failure occurred in the underlying dentine or overlying composite).

### Morphological observations by SEM

After the dentine surface was pretreated with different solution, the root and crown of each tooth were removed along the edge of the wedge-shaped defect using a low-speed diamond saw. Then the specimen was separated along the lowest concave of the wedge-shaped defect and cut perpendicularly to the long axis of teeth into two separate part, each part has a flat surface of dentine. By doing these, the dental wedge-shaped defect of each tooth was divided into specimens with a flat pretreated dentine surface for the scanning electron microscopy (SEM) observation.

All specimens were immersed into neat hexamethyldisilazane solution (HMDS, Electron Microscopy Sciences, Fort Washington, PA, USA) for 30 min, then dried by setting on a filter paper at room temperature for 24 h. After gold-sputter coating of the specimens, the dentine surfaces were observed using SEM (FEI-Quanta 200, FEI, USA) at 20 kV. Three images were taken for each specimen. All of the electron micrographs were taken at the same working distance using the same magnification (2000× and 5000×).

### Statistical analysis

Normality of variable distribution was evaluated with the Kolmogorov-Smirnov test (SPSS 17.0 software; SPSS Inc., Chicago, IL, USA). Then, the micro-tensile bond strength data were statistically analyzed by a one-way ANOVA. Post hoc analysis was performed using Tukey’s test. The level of significance was set at α = 0.05.

## Results

### Micro-tensile bond strength analysis

Micro-tensile bond strength analysis showed that the tension in each group was in the following order: N7 > N8 > N2 > N5 > N3 > N4 > N6 > N1 for the normal dentine, and S8 > S7 > S2 > S4 > S5 > S6 > S1 > S3 for the sclerotic dentine (Table 4). In the normal dentine groups, the highest bond strength was observed in group N7 (*P* < 0.05), and the lowest was observed in group N1 (*P* < 0.05). In the sclerotic dentine groups, the highest bond strength was observed in group S8 (*P* < 0.05), and the lowest was observed in group S3 (*P* < 0.05). In addition, in the normal dentine groups, the bond strengths of N2, N8 and N7 were significantly higher than those of the other groups (*P* < 0.05), and there were no significant differences among that from the groups N2, N8 and N7. In the sclerotic dentine groups, the bond strengths of S8 and S7 were significantly higher than those of the other groups (*P* < 0.05), while there were no significant differences of that between S8 and S7. Moreover, the bond strengths of S2 and S4 were significantly higher than that of S1 (*P* > 0.05), while there was no significant difference of that between S2 and S4. Furthermore, the bond strength of S1 was lower than that of N1, the bond strength of S2 was lower than that of N2, the bond strength of S4 was higher than that of N4 group, and the bond strength of S8 group was significantly higher than that of N8 group (all *P* < 0.05).

**Table 4:** Micro-tensile bond strength values (MPa) (means ± standard deviations) and statistical analysis of all experimental groups

Groups	Pretreatment							
	M1	M2	M3	M4	M5	M6	M7	M8
ND	11.94 ± 0.99^a^	15.82 ± 1.44^b^	12.95 ± 1.38^a^	12.23 ± 1.03^a^	13.40 ± 0.90^a^	12.13 ± 1.03^a^	16.88 ± 1.85^b^	16.42 ± 0.70^b^
SD	11.14 ± 0.71^a^	14.71 ± 0.57^b^	11.04 ± 0.93^a^	14.38 ± 0.91^b^	13.65 ± 0.62^c^	12.92 ± 0.63^d^	19.01 ± 2.55^e^	19.80 ± 1.42^e^
*P* value	< 0.05	< 0.05	< 0.05	< 0.05	> 0.05	> 0.05	> 0.05	< 0.05

Note: M1-M8 represent different pretreatments, as shown in Table 2. *ND* normal dentine; *SD* sclerotic dentine. *n* = 10 for each group. The data are presented as the mean ± SD in MPa. The data with different superscript lowercase letters are significantly different. (*P* < 0.05)

### Fracture pattern analysis

**Fig. 2:**
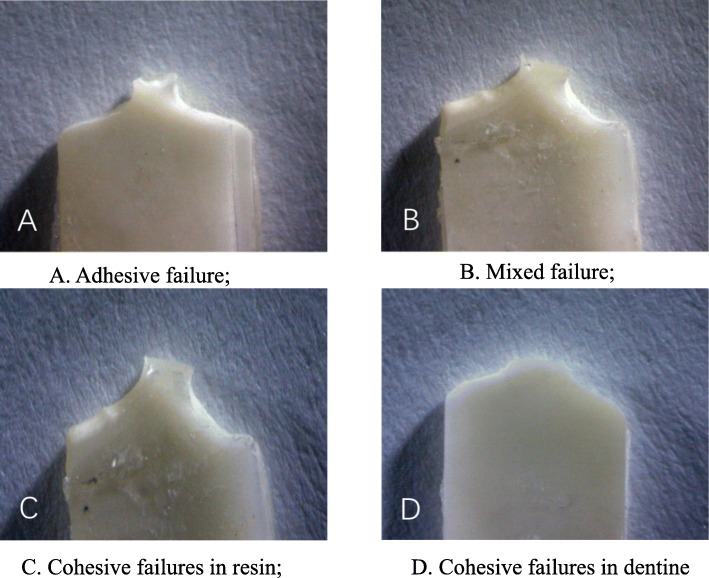
Fracture mode (10×). **a**. Adhesive failure. **b**. Mixed failure. **c**. Cohesive failures in resin, **d**. Cohesive failures in dentine

Fracture pattern observation (Fig. 2) showed that the majority of specimens presented adhesive failures, and a very small portion showed dentine destruction or composite resin failure (cohesive fracture). A small number of mixed failures were observed.

Statistical analysis showed that there were no significant differences in the fracture mode frequency of sclerotic and normal dentine using different pretreatment methods (*P* > 0.05). The fracture pattern of each group is shown in Table 5.

**Table 5:** Failure mode frequency (number)

Group	Number of samples	Fracture mode		
		Adhesive failure	Mixed failure	Cohesive failure
N1	10	10	0	0
S1	10	10	0	0
N2	10	9	0	1
S2	10	7	0	3
N3	10	10	0	0
S3	10	7	0	3
N4	10	10	0	0
S4	10	10	0	0
N5	10	10	0	0
S5	10	10	0	0
N6	10	10	0	0
S6	10	9	0	1
N7	10	10	0	0
S7	10	10	0	0
N8	10	9	1	0
S8	10	8	1	1

Note: N1-N8 represent normal dentine groups, S1-S8 represent sclerotic dentine groups

### Morphological changes of the dentine surface

**Fig. 3:**
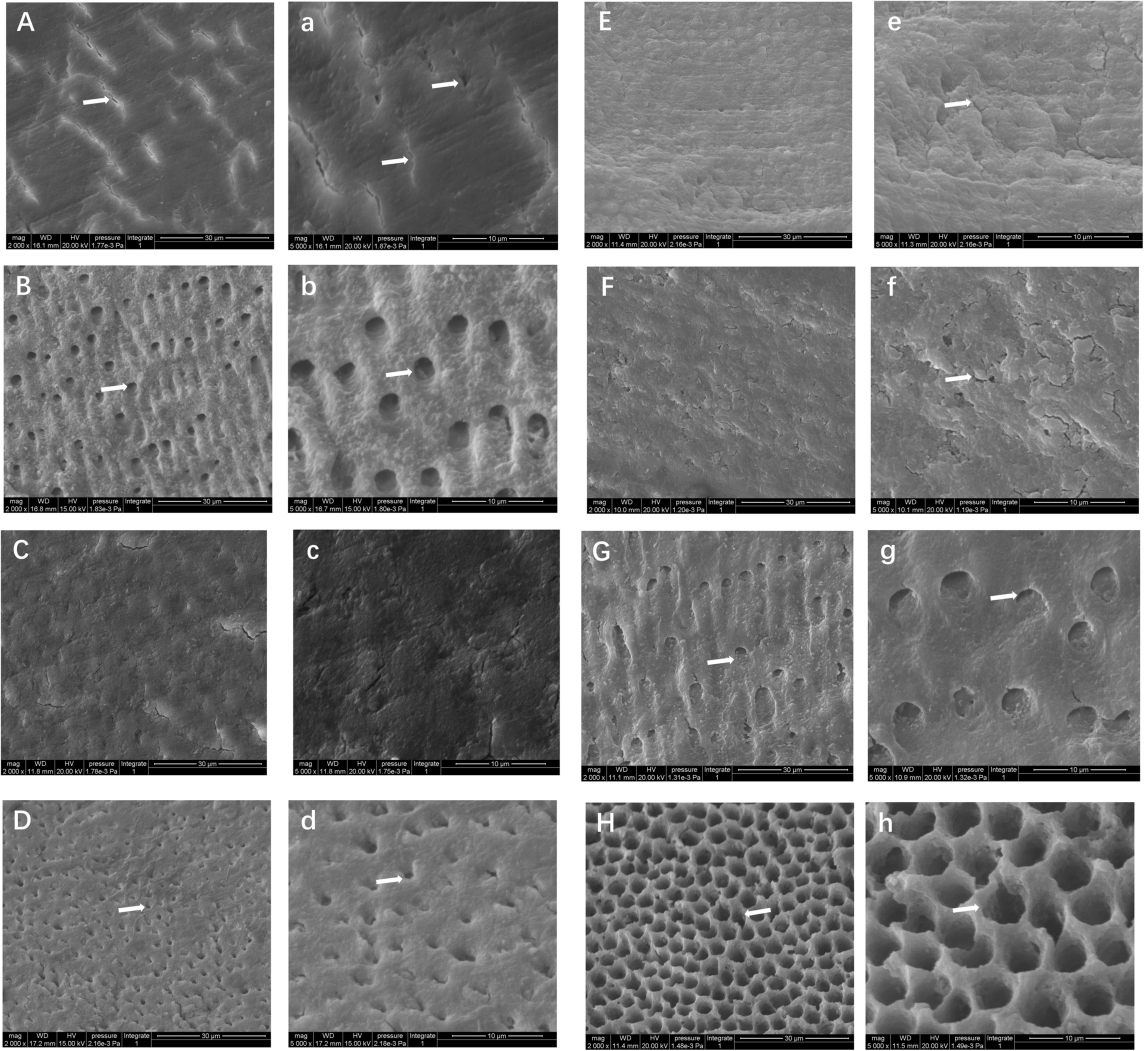
SEM micrographs of normal dentine surfaces (magnification, 2000× and 5000×). A × 2000, a × 5000: Control group and normal dentine without any treatments (a smear layer and a few cracks were observed (arrows)); B × 2000, b × 5000: dentine after 35% phosphoric acid treatment (dentine tubules were exposed after the treatment (arrows)); C × 2000, c × 5000: dentine after 5% NaClO treatment (the surface did not exhibit any obvious changes); D × 2000, d × 5000: dentine after 15% EDTA treatment (demineralized holes were observed (arrows)); E × 2000, e × 5000: dentine after 15% EDTA+ 5% NaClO treatment (an increased surface roughness was observed); F × 2000, f × 5000: dentine after 15% EDTA+ 10% NaClO treatment (an increased surface roughness was observed); G × 2000, g × 5000: dentine after 35% phosphoric acid+ 5% NaClO treatment (larger-diameter dentinal tubules and deeper holes were observed); H × 2000, h × 5000: dentine after 35% phosphoric acid+ 10% NaClO treatment (linked network structure was observed)

**Fig. 4:**
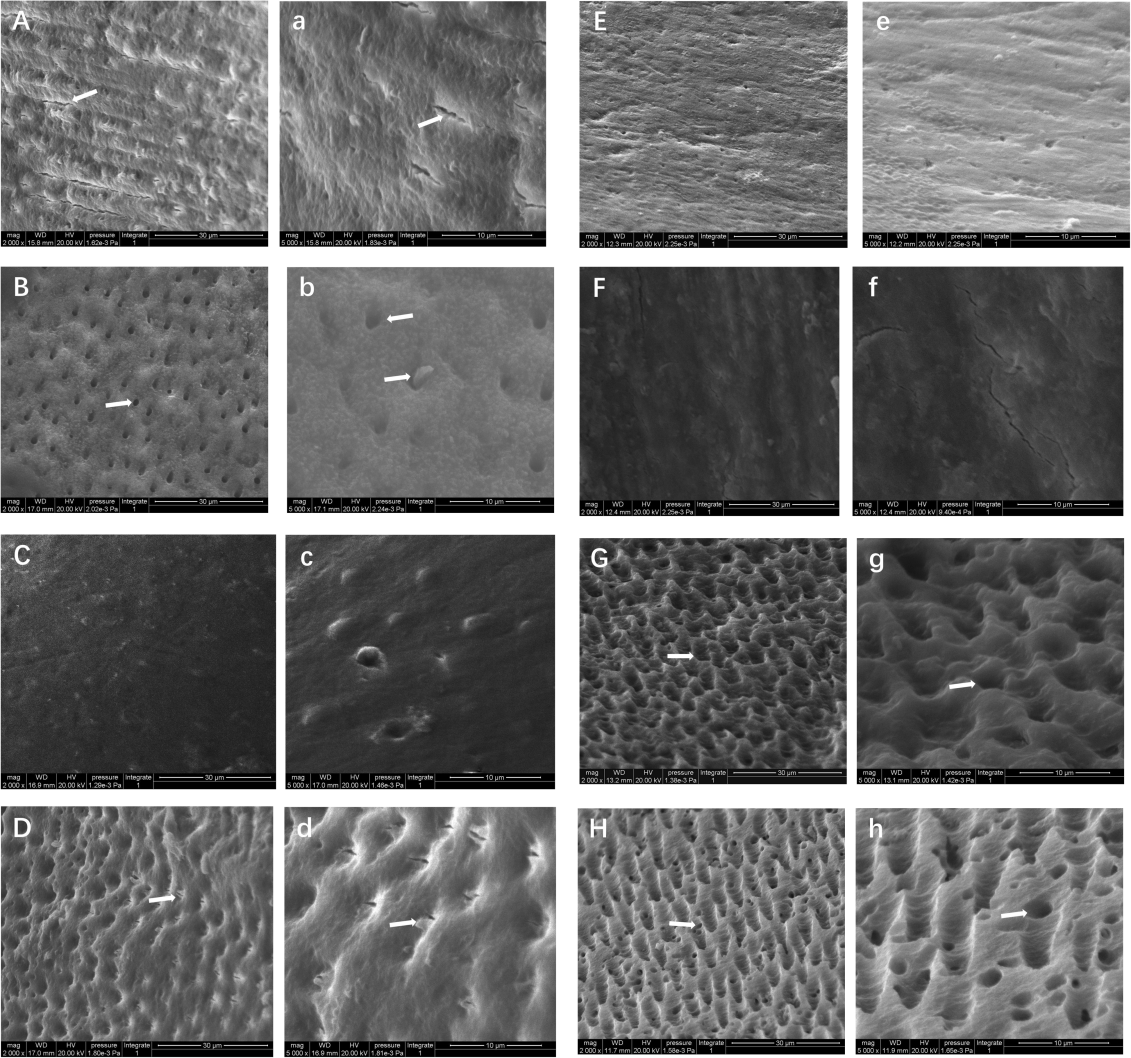
SEM micrographs of sclerotic dentine surfaces (magnification, 2000× and 5000×). A × 2000, a × 5000: Control group and sclerotic dentine without any treatments (shallow grooves were observed (arrows)); B × 2000, b × 5000: dentine after 35% phosphoric acid treatment (sclerotic casts extending from open dentine tubules are shown (arrows)); C × 2000, c × 5000: dentine after 5% NaClO treatment (the surface did not exhibit any obvious changes); D × 2000, d × 5000: dentine after 15% EDTA treatment (demineralized holes and peritubular dentine were observed (arrows)); E × 2000, e × 5000: dentine after 15% EDTA+ 5% NaClO treatment (an increased surface roughness was observed); F × 2000, f × 5000: dentine after 15% EDTA+ 10% NaClO treatment (an increased surface roughness was observed); G × 2000, g × 5000: dentine after 35% phosphoric acid+ 5% NaClO treatment (a rough surface similar to that of normal dentine in N7 group); H × 2000, h × 5000: dentine after 35% phosphoric acid+ 10% NaClO treatment (a clear and rugged surface was observed)

SEM observation showed that the surface of normal dentine (artificial lesion) without any pretreatment was covered with a smear layer and a few cracks (Fig. 3-A, 3-a). In contrast, the surface of the sclerotic dentine (natural lesion) exhibited a smear layer with shallow grooves and only a few visible dentinal tubule orifices (Fig. 4-A).

In groups N2 and S2, after 35% phosphate etching pretreatment, the surfaces of both the sclerotic and the normal dentine appeared with the open dentinal tubules or the demineralized holes as compared to that of the un-treated control groups, respectively (Fig. 3-B and Fig. 4-B). The dentinal tubules on the surface of the pretreated normal dentine (Fig. 3-b) had larger diameters and deeper holes than those of the pretreated sclerotic dentine groups (Fig. 4-b). Moreover, sclerotic cast extrusion from the dentinal tubules was also observed in the pretreated sclerotic dentine groups (Fig. 4-b).

In groups N3 and S3, after 5% NaClO pretreatment, the surfaces of both the normal and the sclerotic dentine did not show any obvious changes, and the tubules remained blocked compared to those on the surface of the un-treated control dentine groups (Fig. 3-C and 4-C), respectively.

In groups N4 and S4, after 15% EDTA pretreatment, the surfaces of both sclerotic and normal dentine appeared with the open dentine tubules or the demineralized holes as compared to that of the un-treated control groups (Fig. 3-D and 4-D), respectively. Moreover, there are more peritubular dentine in the pretreated sclerotic dentine than that in the pretreated normal dentine (Fig. 3-d and 4-d).

In groups N5 and S5 (after pretreatment of 15% EDTA+ 5% NaClO), and groups N6 and S6 (after pretreatment of 15% EDTA+ 10% NaClO), the surfaces of both sclerotic and normal dentine presented with an increased surface roughness as compared to that of the un-treated control groups (Fig. 3-E and F; Fig. 4-E and F), respectively.

In groups N7 and S7 (after 35% phosphoric acid+ 5% NaClO), and groups N8 and S8 (after pretreatment of 35% phosphoric acid+ 10% NaClO), the surface of both sclerotic and normal dentine appeared with open dentine tubules or demineralized holes compared to that of the untreated group, respectively (Fig. 3-G and 4-G). After 35% phosphoric acid+ 5% NaClO pretreatment of N7, the surface of normal dentine appeared with larger-diameter dentine tubules and deeper holes (Fig. 3-g) than that of the sclerotic dentine (Fig. 4-g). After 35% phosphoric acid+ 10% NaClO pretreatment of N8, the normal dentine presented the most irregular surface, exhibiting a linked network structure, while the sclerotic dentine had a similar superficial aspect, with a clear, rugged surface and more irregular inter-tubular dentine (Fig. 3-H and 4-H).

## Discussion

Sclerotic dentin [7] is a clinically relevant multilayered bonding substrate in which the dentine has been physiologically and pathologically altered, partly as the body’s natural defence mechanism to injury, and partly as a consequence of colonization by oral microflora growth in the layer [7]. At an ultra-structural level, sclerotic dentine in natural cervical wedge-shaped lesions showed tiny, electron-dense crystallites surrounded by a tube-like membranous structure [18, 26], which instead of a normal tubular occlusion. The presence of a hyper-mineralized surface layer, bacteria, mineralized bacterial matrices and sclerotic casts obliterates the dentinal tubules, makes sclerotic cervical dentine less susceptible to acid demineralization, and also reduces the acid corrosion depth and penetration depth of the resin. This substrate has been demonstrated to be a challenge for bonding procedures [10–13].

The natural cervical wedge-shaped lesions with no caries is a kind of sclerotic dentine. Because many in vitro studies [4, 5] have demonstrated that sclerotic dentine provides a lower bond strength than normal dentine due to acid conditioning resistance [7, 27], some clinicians would like to remove the surface layer of the cervical wedge-shaped lesions [26]. However, other studies have instructed clinicians to avoid cavity preparation under this circumstance, particularly with the use of a medium-grit diamond bur [17, 28]. Moreover, Kusunoki [29] et al. considered that sclerotic dentine represented a natural protective mechanism of the body and should be preserved. At present, no clear procedure has been found to ensure the adhesive efficacy of sclerotic dentine of the human teeth. Other clinical approaches should be investigated to improve the bond strength of self-etch systems and sclerotic dentine of human teeth.

In recent years, some scholars have proposed the concept of surface pretreatment, which pretreats the surface of hardened dentine before adhesion. Clinical pretreatment agents included EDTA, citric acid, oxalic acid, and povidone-iodine [30, 31] in the previous studies, while the comparisons between each two of the different pretreatment agents such as phosphoric acid, NaClO, EDTA, and the effects of the combinations of those two agents on dentine adhesion of the human teeth are still lacking. This study evaluated the effects of different surface treatment agents on the micro-tensile bond strength and microstructure of sclerotic dentine and normal dentine.

The most frequently used tests to evaluate the bonding effectiveness of adhesive systems are bond strength tests, such as the micro-tensile bond strength. In 1994, Sano [30, 31] et al. put forward the micro-tensile experimental method. The major advantage of the TBS test is ascribed to the measurement of the bond strength of relatively small specimens (< 1 mm^2^ cross-sectional areas). Although the TBS test is more complicated, operator-sensitive and time-consuming, this test still apply to this study. Another advantage of the TBS test is the uniform stress strain field, which is important to achieve most of the failure on the bond interface [32, 33]. In addition, many studies have addressed the application of micro-tensile experiments, which is more applicable for the detection of bonding strength due to neck wedge defects [22, 34, 35].

The results showed that pretreatment with 35% phosphoric acid conditioning could increase the adhesive ability for both sclerotic and normal dentine. The morphological observation consistent with the results of micro-tension bonding strength test, which suggested that the adjunctive phosphoric acid pre-conditioning could diminish the peritubular dentine and contribute to the increased bond strength between the resin and dentine. Previous studies have shown an increase in μ-TBS of Single Bond to caries-affected hyper-mineralized sclerotic human dentine and cervical sclerotic human dentine when the acid etching time was extended to 30 s [36]. The use of adjunctive phosphoric acid preconditioning has been suggested as a means to improve the bonding of self-etching primers to sclerotic dentine with thick smear layers. This study was consistent with the previous studies, which suggested that the adjunctive use of an acid solution on ground sclerotic dentine before applying a self-etch adhesive will probably be constructive.

Previous research has demonstrated that EDTA pretreatment can improve the bonding strength of normal dentine and sclerotic dentine [37, 38]. But, Torii et al. [39], who used EDTA instead of phosphoric acid to condition normal bovine dentine before applying the Single Bond, which showed that the procedure did not result in an increase in mean bond strength values. In this study, the results showed that EDTA pretreatment could improve the self-acid bonding strength of sclerotic dentine of human teeth, while no obvious effect was found for the normal dentine. In contrast, SEM observation showed a wider opening of the tubules in both normal and sclerotic dentine subjected to 15% EDTA pretreatment. The above results may be due by the special demineralization caused by EDTA and the physiological and pathological differences of the dentine. First, the organic compound EDTA is a mild chelating agent with 4 carboxylic acid groups, which can chelate calcium ions and selectively remove hydroxyapatite without entering deeply into the dentine tubule, as well as maintain the structure of the collagen matrix without collagen denaturation. Second, the hyper-mineralized layer of sclerotic dentine exhibits a high degree of variability compared with normal dentine, both in terms of dentinal tubule obliteration by mineral salts and thickness of the surface hyper-mineralized layer [7], which prevent hybridization of the underlying sclerotic dentine [9–13]. The different effects of EDTA on sclerotic and normal dentine in this study suggested that EDTA exerted a mild demineralization on the peritubular dentine of sclerotic dentine, maintaining the high calcium content and gradually opening the tubules, which are advantageous features of the chemical combination of calcium with the functional monomer of resin and the mechanical combination of dentin and resin. Thus, the bond strength of sclerotic dentine increased after EDTA pretreatment in the study.

Further, SEM observation revealed there were no open tubules on the surface of both normal and sclerotic dentine after pretreatment with 15% EDTA+ 5% NaClO and 15% EDTA+ 10% NaClO, which could explain the absence of an obvious improvement of the bonding strength of dentine after that pretreatment, as compared to that of the non-pretreated controls, regardless of the kind of dentine.

The most important results in the present study were that pretreatment of normal and sclerotic dentine with 35% phosphoric acid+ 5% or 10% NaClO significantly improved the bonding strength using a self-etching adhesive system, as compared to the non-pretreated controls. And, after 35% phosphoric acid+ 5% or 10% NaClO pretreatment, the bonding strength of sclerotic dentine was significantly greater than that of normal dentine (*P* < 0.05). Furthermore, SEM observation showed that the sclerotic dentine pretreated with 35% phosphoric acid+ 5% or 10% NaClO had a similar superficial aspect to that of the normal dentine with a large number of open tubules, meanwhile the smear layer, peritubular dentine and etched exposed collagen fibres were removed after these pretreatments. The SEM images showed a clear exposure of the tubular collateral net of the dentine. The results suggested that the application of 35% phosphoric acid conditioning to dentine could demineralize the collagen fibres and decalcify the superficial dentine layer, and subsequently 10% or 5% NaClO could dissolve and remove the exposed dentinal collagen, providing a fresh dentine apatite surface for the applied adhesive resin [40]. This process allowed direct adhesion between the adhesive resin and dentine, with no resin-reinforced layer of dentinal collagen and the adhesive resin between the two layers [41]. Above all, the results indicate that pretreatment of dentine using 35% phosphoric acid+ 5% or + 10% sodium hypochlorite could alter the microstructure of the sclerotic dentine surface and improve the micro-tensile bond strength, which is opposed to the null hypothesis at the beginning. The effects of the pretreatment and subsequent adhesion might be determined by the demineralized extent of the surface and the relevant characteristics of the substrate of the dentine. The relevant mechanisms of the different pretreatments need to be pursued in the future study.

## Conclusion

Pretreatment of dentine using 35% phosphoric acid+ 5% sodium hypochlorite, and 35% phosphoric acid+ 10% sodium hypochlorite changed the surface of the sclerotic dentine and subsequently increased the micro-tensile bond strength.

## Data Availability

The datasets used and analyzed during the current study available from the corresponding author on reasonable request.

## Abbreviations

EDTA: Ethylene Diamine Tetraacetic Acid
HMDS: Hexamethyldisilazane solution
IRB: Institutional Review Board
ND: Normal dentine
SD: Sclerotic dentine
SEM: Scanning electron microscopy
μ-TBS: Micro-tensile test
